# Ginseng Rb Fraction Protects Glia, Neurons and Cognitive Function in a Rat Model of Neurodegeneration

**DOI:** 10.1371/journal.pone.0101077

**Published:** 2014-06-27

**Authors:** Kangning Xu, Yufen Zhang, Yan Wang, Peng Ling, Xin Xie, Chenyao Jiang, Zhizhen Zhang, Xiao-Yuan Lian

**Affiliations:** 1 College of Pharmaceutical Sciences, Zhejiang University, Hangzhou, China; 2 Ocean College, Zhejiang University, Hangzhou, China; 3 Anhui University of Chinese Medicine, Hefei, China; University of Modena and Reggio Emilia, Italy

## Abstract

The loss and injury of neurons play an important role in the onset of various neurodegenerative diseases, while both microgliosis and astrocyte loss or dysfunction are significant causes of neuronal degeneration. Previous studies have suggested that an extract enriched panaxadiol saponins from ginseng has more neuroprotective potential than the total saponins of ginseng. The present study investigated whether a fraction of highly purified panaxadiol saponins (termed as Rb fraction) was protective for both glia and neurons, especially GABAergic interneurons, against kainic acid (KA)-induced excitotoxicity in rats. Rats received Rb fraction at 30 mg/kg (ip), 40 mg/kg (ip or saline followed 40 min later by an intracerebroventricular injection of KA. Acute hippocampal injury was determined at 48 h after KA, and impairment of hippocampus-dependent learning and memory as well as delayed neuronal injury was determined 16 to 21 days later. KA injection produced significant acute hippocampal injuries, including GAD67-positive GABAergic interneuron loss in CA1, paralbumin (PV)-positive GABAergic interneuron loss, pyramidal neuron degeneration and astrocyte damage accompanied with reactive microglia in both CA1 and CA3 regions of the hippocampus. There was also a delayed loss of GAD67-positive interneurons in CA1, CA3, hilus and dentate gyrus. Microgliosis also became more severe 21 days later. Accordingly, KA injection resulted in hippocampus-dependent spatial memory impairment. Interestingly, the pretreatment with Rb fraction at 30 or 40 mg/kg significantly protected the pyramidal neurons and GABAergic interneurons against KA-induced acute excitotoxicity and delayed injury. Rb fraction also prevented memory impairments and protected astrocytes from KA-induced acute excitotoxicity. Additionally, microglial activation, especially the delayed microgliosis, was inhibited by Rb fraction. Overall, this study demonstrated that Rb fraction protected both astrocytes and neurons, especially GABAergic interneurons, and maintained microglial homeostasis against KA-induced excitotoxicity. Therefore, Rb fraction has the potential to prevent and treat neurodegenerative diseases.

## Introduction

Brain diseases in humans are almost universally attributed to the malfunction or loss of nerve cells [1]. Excitotoxicity involving the excitatory glutamate receptors is a key cause of acute neuronal damage in traumatic brain injury, stroke, and various neurodegenerative disorders such as Alzheimer's disease, Parkinson disease, epilepsy, and seizures [2]. The impairment of glutamate reuptake by astrocytes and GABAergic cells can lead to extracellular glutamate accumulation, inhibition weakness, and, consequently, neuronal excitotoxicity [3]. While GABAergic interneurons, particularly certain important subpopulations such as ones containing the calcium binding protein parvalbumin (PV) in the cortex and hippocampus, have been shown to be very vulnerable to excitotoxicity [4]–[6], astrocytes could be even more susceptible to neurotoxic insults. For example, ischemia has been shown to cause a sequential impairment to cortical astrocytes and GABAergic neurons, and the excitotoxicity due to this impairment of astrocytic functions contributes to GABAergic cell death [7]. Microglial activation has also been shown to be another important contributor to excitotoxicity [8]. Therefore, astrocyte loss or dysfunction, microglial activation, GABAergic injury and excitotoxicity can form a vicious cycle. Interestingly, this cycle seems to exist in the process of neurodegeneration induced by kainic acid (KA), a potent agonist of α-amino-3-hydroxy-5-methyl-4- isoxazolepropionic acid (AMPA)/kainate glutamate receptors. Systemic or intracerebroventricular injection of KA can induce seizures [9], [10] and lead to neurodegeneration in many regions of the brain in rodents, particularly in the hippocampal subregions of CA1 and CA3, and in the hilus of dentate gyrus (DG) [11]. Therefore, KA has been widely used to study the mechanisms of neurodegeneration induced by excitotoxicity and to discover new neuroprotective agents [8], [11], [12]. More recent studies indicated that GABAergic interneurons, particularly PV+ cells, are highly susceptible to KA toxicity [4], [6], [13]. In addition to damaging neurons, KA can strongly activate astrocytes and microglia, thus leading to inflammatory environments [14], [15], which has been shown to be involved in KA-induced neuron death, especially delayed neurodegeneration [8], [16], [17]. In this context, it is noteworthy that deficits in GABAergic interneurons are implicated in multiple psychiatric and neurological disorders, such as schizophrenia, epilepsy and intellectual disability in AD [18]–[22]. In particular, hippocampal PV+ interneurons in the cortex and the hippocampus innervate hundreds of pyramidal neurons mainly at the soma and proximal dendrites, control these neurons' output and synchrony [23]–[25], and thus contribute to the generation of the gamma-frequency oscillations that has been believed to be important for cognitive functions, such as memory formation and sensory processing [26]. Consistently, an impairment of hippocampal PV+ interneurons has been shown to be responsible for cognitive deficits in AD mice [18], [19], [22]. Taken together, simultaneous protection of astrocytes and GABAergic interneurons while maintaining glial homeostasis is crucial for the prevention or treatment of neurodegenerative disorders.

Ginseng, a famous traditional Chinese medicine, has been widely used as a tonic and restorative agent by Asians for thousands of years and is now a popular natural medicine used worldwide. It is well known that ginsenosides, also called ginseng saponins, are the major bioactive components of ginseng [27]. Ginsenosides are divided into two major groups of panaxadiol saponins (PDS) and panaxatriol saponins (PTS). Our previous studies have demonstrated that a purified PDS extract (mainly contained panaxadiol saponins of ginsenosides Rb_1_, Rb_3_ and Rd, termed Rb extract) is much more effective than the total ginsenosides (PDS plus PTS) in animal models of 3-nitropropionic acid -induced neurodegeneration and chemical convulsants-induced seizures while the ginseng crude extract has not been proven to be effective [28]–[30]. These results suggest that the Rb extract is more neuroprotective than the total saponins of ginseng. In this context, one could predict that a fraction containing more individuals of PDS should be more effective than the Rb extract. To the best of our knowledge, it is unclear whether the Rb extract is able to maintain glial homeostasis or protect astrocytes and GABAergic neurons. Therefore, this study aims to investigate whether a highly purified PDS fraction (termed as Rb fraction) is protective for both glia and neurons, particularly GABAergic interneurons, against KA-induced excitotoxicity in rats. The data indicate that this Rb fraction can protect GABAergic interneurons, particularly PV+ subtype, and principle neurons in the hippocampus, and then consequently preserve cognitive function against KA-induced excitotoxicity. The Rb fraction can also protect astrocytes and inhibit microglial activation, which partially contributes to the neuroprotective actions of the Rb fraction.

## Materials and Methods

### Ethics Statement

This study was carried out in strict accordance with the recommendations in the Guide for the Care and Use of Laboratory Animals of the National Institutes of Health. All experimental protocols were approved by the Institutional Animal Ethics Committee of Zhejiang University. All surgery was performed under sodium pentobarbital anesthesia, and all efforts were made to minimize any pain or discomfort, and the minimum number of animals was used.

### Animals

**Figure 1:**
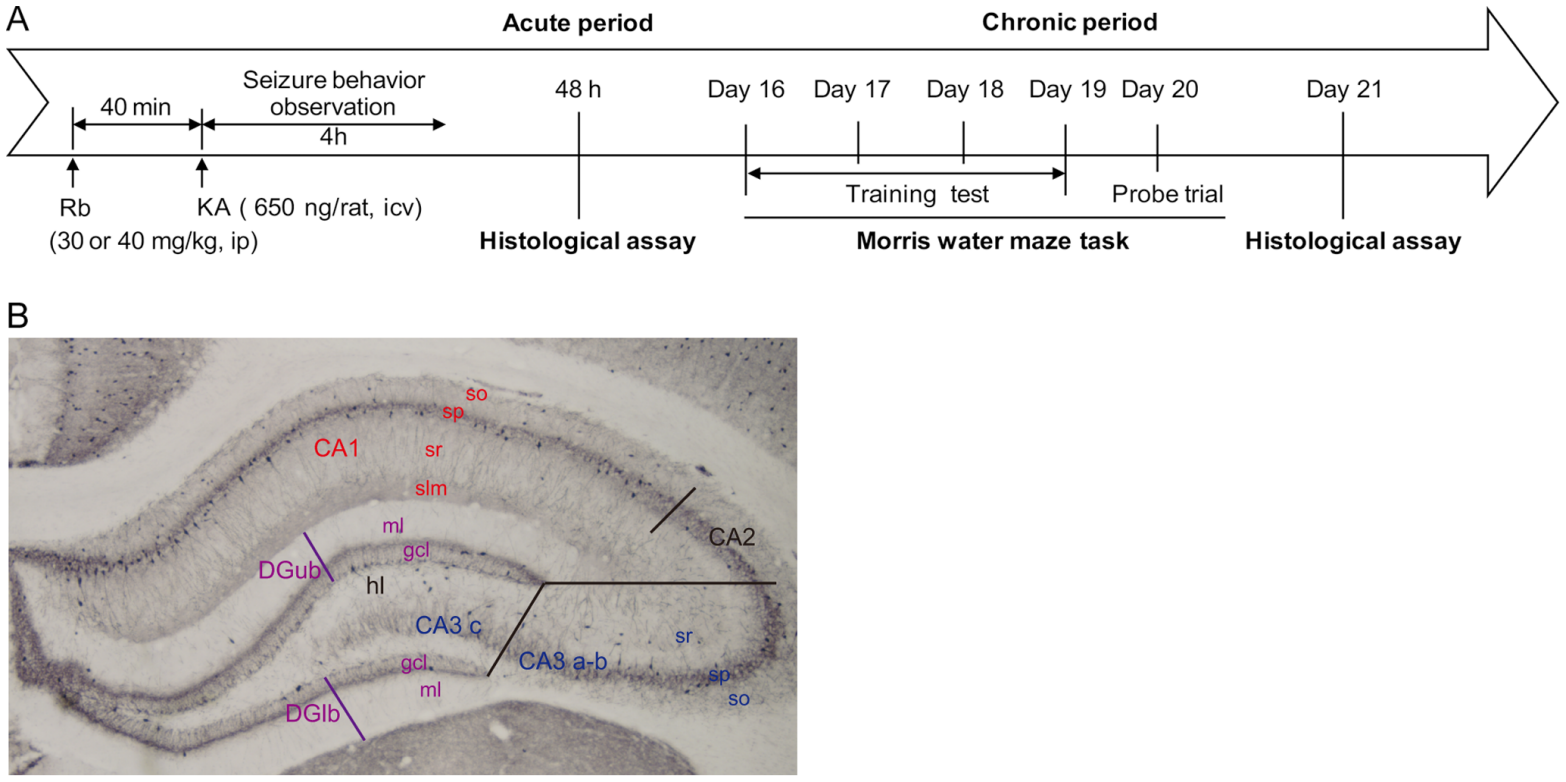
Some instructions for the methods. (A) Experimental design. Rb fraction (Rb) was administrated intraperitoneally 40 min before intracerebroventricular (icv) KA injection. Rats were sacrificed at 48 h (acute period) or 3 weeks (chronic period) after KA for histological assay. The Morris water maze task was conducted from the 16^th^ to 19^th^ day for the training test and the 20^th^ day for the probe trial after KA injection. (B) Division of the dorsal hippocampus. The CA2 subfield is small and difficult to separate from the CA1, and thus was included in the CA1 subfield. so, stratum oriens; sp, stratum pyramidale; sr, stratum radiatum; slm, stratum lacunosum-moleculare; DGub, dentate gyrus upper blade; DGlb, dentate lower blade; gcl, granule cell layer; ml, molecular layer; hl, hilus.

Male Sprague-Dawley rats (220–240 g, Experimental Animal Center, Medical Science Academy of Zhejiang Province, Hangzhou, China) were housed under controlled conditions with food and water available *ad libitum*. Animals were randomly assigned to a KA-treated, KA plus Rb fraction, or saline control group, with 6–8 animals in each group. The experiment design is shown in Fig. 1A.

### KA stereotaxic injection

Prior to surgery, rats were anesthetized with pentobarbital sodium (50 mg/kg, i.p.) and positioned in a stereotaxic instrument. A drill hole was made in the skull above the left side of the lateral ventricle (1.0 mm posterior to bregma, 1.5 mm lateral to the midline). KA (Sigma-Aldrich) of a concentration of 100 ng/µL in 0.01 M phosphate buffered saline (PBS, pH 7.4) was administered into the lateral ventricle (AP -1.0 mm, lateral 1.5 mm; ventral −3.8 mm from the skull) at a volume of 6.5 µL via a 28-gauge stainless steel needle attached to a 10 µL Hamilton syringe. The injection needle was left in place for an additional 2 min to prevent back diffusion of the injected solution. Another set of animals was injected with the same volume of saline as saline controls (Saline). After the injection, rats were kept on a warm pad until awake and then placed in plastic cages for observation of seizure activity.

### Assessment of seizure behavior

Once rats awakened after KA administration, seizure behaviors were observed for 4 hours by a person blind to the treatment group and scored using the following scale [31]:(+1) arrest of motion; (+2) myoclonic jerk of head and neck with brief twitching movements; (+3) unilateral clonic activity, frequent focal convulsions, salivation; (+4) bilateral forelimb tonic and clonic activity, frequent focal convulsions; and (+5) continuous generalized limbic seizures with loss of postural tone, or death within 4 hours. Twenty four and forty eight hours later, rats were weighed to assess weight loss, which has been shown to positively correlate with seizure severity and duration [30]. In our previous study, animals who had ≥ stage 3 seizures or ≥ 10% weight loss 24 hours later following KA administration also had robust neuronal damage in the hippocampus after KA [29]. Thus, the animals who had only mild seizures (lower than stage 3 seizures, or brief stage 3 and higher stage seizures) or a body weight loss lower than 10% in all the experimental groups were excluded for the later experiments.

### Drug treatment

Rb fraction was prepared in our own laboratory using the reported method [32] and was composed of 91.95% panaxadiol saponins including five ginsenosides Rb1, Rb2, Rb3, Rc, and Rd. The Rb fraction was dissolved in normal saline and administered intraperitoneally once 40 min before KA injection. Two doses of Rb fraction (30 and 40 mg/kg) were tested with this dosing schedule. These two doses were chosen according to our previous studies [28]–[30] in which the dose range of 10–40 mg/kg was demonstrated to be effective, with 30–40\ mg/kg being the most effective. Control animals received the same volume of saline.

### Morris Water Maze: Training test and probe trial

On the 16^th^ day post-KA, cognitive function was determined using the Morris water maze previously described [33] with little modifications. The Morris water maze task was conducted in a circular 150-cm-diameter pool with a black-painted inner surface. The pool was filled with water to a depth of 31.5 cm (maintained at 20.0 ± 1.0°C). A circular platform (10 cm) was submerged 2 cm below the water level and was located in a fixed position in the middle of one quadrant, equidistant from the center and edge of the pool. The pool was surrounded by several distal visual cues. In the training test, the rats were given 4 trials per day for 4 consecutive days starting counterclockwise from one of the four quadrants each time. The animals were allowed to swim for 120 s and then allowed to rest on the platform for 10 s after mounting the platform. If the rats failed to find the platform within 120 s, they were guided onto the platform and allowed to rest on the platform for 10 s. The time taken to reach the platform was measured as escape latency in each of the four trials for all animals. Twenty-four hours later, we conducted a probe trial in which the platform was removed and the rat was allowed to swim for 90 s. The trial started with the rat in the quadrant opposite to the trained platform location. The numbers of times they swam across the area where the platform had been located were recorded.

### Animal perfusion and tissue processing

Forty eight hours or 3 weeks after KA injection, animals were deeply anesthetized with pentobarbital sodium (60 mg/kg, i.p.) and transcardially perfused with saline followed by 4% paraformaldehyde in 0.01 M PBS. Brains were removed and postfixed in the same fixative. Coronal brain sections were cut at 30 µm using a Vibratome (Leica, Germany). Sections of the dorsal hippocampus from approximately 2.4 to 3.6 mm posterior to bregma were collected [34]. Every ten sections, six sections were set aside for staining with a total of four sets (twenty-four sections) for each animal. The first section of each set was stained with Fluoro-Jade C (FJC, Histochem, Jefferson, AR, USA), the second section was stained with cresyl violet, and the third to sixth sections were stained for immunohistochemistry of GAD67, PV, GFAP, and Iba-1, respectively.

### FJC staining and Nissl staining

FJC staining that can identify dying neurons was conducted to evaluate neurodegeneration after KA injection according to FJC kit instructions. Brain sections mounted on the gelatin-coated glass slide were immersed into an 80% alcohol solution containing 1% NaOH for 5 minutes, and then into 70% ethanol for 2 minutes. After being washed in water, the slides were submerged in 0.06% KMnO_4_ for 10 minutes, washed again in water, and finally incubated in a solution composed of 0.001% FJC (in 0.1% acetic acid) for 20 minutes.

To confirm the results from FJC staining, the adjacent sections to the FJC stained ones were stained with cresyl violet to identify survival neurons. Briefly, brain sections were mounted onto gelatin-coated glass slides and allowed to dry for 3 hours. Once dry, the sections were rehydrated in distilled water, incubated in 0.1% cresyl violet solution (Sigma-Aldrich) for 15-30 minutes until the desired depth of staining was achieved. The sections were then dehydrated through a series of graded ethanol solutions of increasing concentrations (50%, 95%, 100%) and cleared in xylenes before they were coverslipped using Neutral balsam.

### Immunohistochemistry

GABAergic interneurons were identified by PV or glutamic acid decarboxylase 67 (GAD67), which exists in all GABAergic neurons and is the rate-limiting enzyme for the synthesis of GABA [35], and astrocytes and microglia were identified by GFAP and Iba-1, respectively, using immunohistochemistry. Briefly, after heating antigen retrieval (incubating the sections at 98°C in a Tris-EDTA buffer for 5 minutes), sections were incubated in 3% H_2_O_2_ for 20 minutes to quench endogenous peroxidase activity. The sections were then washed three times in PBST (PBS containing 0.25% Triton-100X), and incubated in 2% bovine serum albumin to block non-specific immunoreactivity. After blocking, the sections were incubated with anti-PV (1:4000, mouse monoclonal antibody, Abcam), anti-GAD67 (1:10000, mouse monoclonal antibody, Abcam), anti-GFAP (1:2000, rabbit polyclonal antibody, Abcam) or anti-Iba-1 (1:2000, rabbit polyclonal antibody, Wako) overnight at 4°C, followed by goat anti-mouse or anti-rabbit antibody conjugated with horseradish peroxidase (PV-9000 Kit; Zhong-shan-jin-qiao biotechnology), and then visualized with diaminobenzidine.

### Imaging and Quantification

Photo images of the entire hippocampus in both right and left hemispheres from the 6 to 8 rats per group (four sections per rat) were obtained at 40× magnification using a Nikon microscope equipped with a laser lamp. Sections stained with FJC were observed to determine neuronal injury. The severity of neurodegeneration in the pyramidal cell layers in the whole dorsal CA1 and CA3 (CA3a-b and CA3c) (Fig. 1B) areas in both brain hemispheres was scored by a person blind to the treatment group using the 0–3 scale: 0, no observable FJC positive staining; 1 (slightly injured), the area with the positive staining less than one third area of the target region; 2 (moderately injured), the half area of the target region stained; 3 (severe injury), more than two thirds area of the target region stained. Damage scores from both hemispheres were added together and then averaged across the four sections for each rat.

To assess the numbers of GAD67+ cells, PV+ cells, astrocytes or microglia in the hippocampus, images captured by the microscope were saved as tiff files, then magnified 1000× on a computer screen to facilitate cell count. The assayed areas of the hippocampus for GAD67+ or PV+ cells included the whole dorsal CA1 subfield (across the layers of stratum oriens, pyramidal cell layer, stratum radium and lacunosum-moleculare), CA3a-b subfield (including stratum oriens, pyramidal cell layer and stratum radium), hilus (also including CA3c), dentate gyrus (across the molecular and granule cell layers of both upper blade (DGub) and lower blade (DGlb) (Fig. 1B) in both hemispheres. For astrocytes or microglia, the assayed areas included the whole dorsal CA1 stratum radium and the CA3a-b stratum radium. This cell count was conducted by an observer blind to the treatment group, and the cell numbers from both hemispheres were added together and then averaged across the four sections for each rat.

### Statistical Analysis

All data are expressed as means ± standard error of the mean (SEM). Group differences in the escape latency in the Morris water maze task were analyzed using a two-way analysis of variance (ANOVA) with repeated measures, the factors being treatment and testing day during the training test. Otherwise, data were analyzed by a one-way ANOVA followed by Dunnet's post-test. *P* < 0.05 was considered statistically significant.

## Results

### Rb fraction suppresses KA-induced acute neurodegeneration in hippocampus

**Figure 2:**
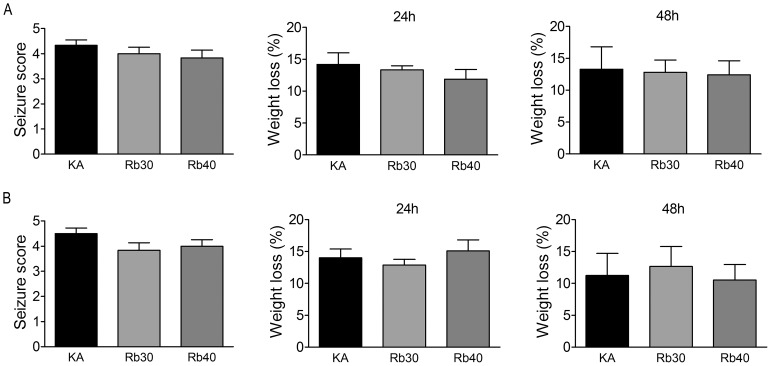
Seizure score and body weight loss after KA administration. Seizure score and body weight loss at 24-induced acute hippocampal damage (A) and in the animals used to determine delayed neurodegeneration during chronic period (B) after KA injection. Data are expressed as means ± SEM and analyzed by a one-way analysis of variance followed by Dunnet's post-test; n  =  6–8 per group.

**Figure 3:**
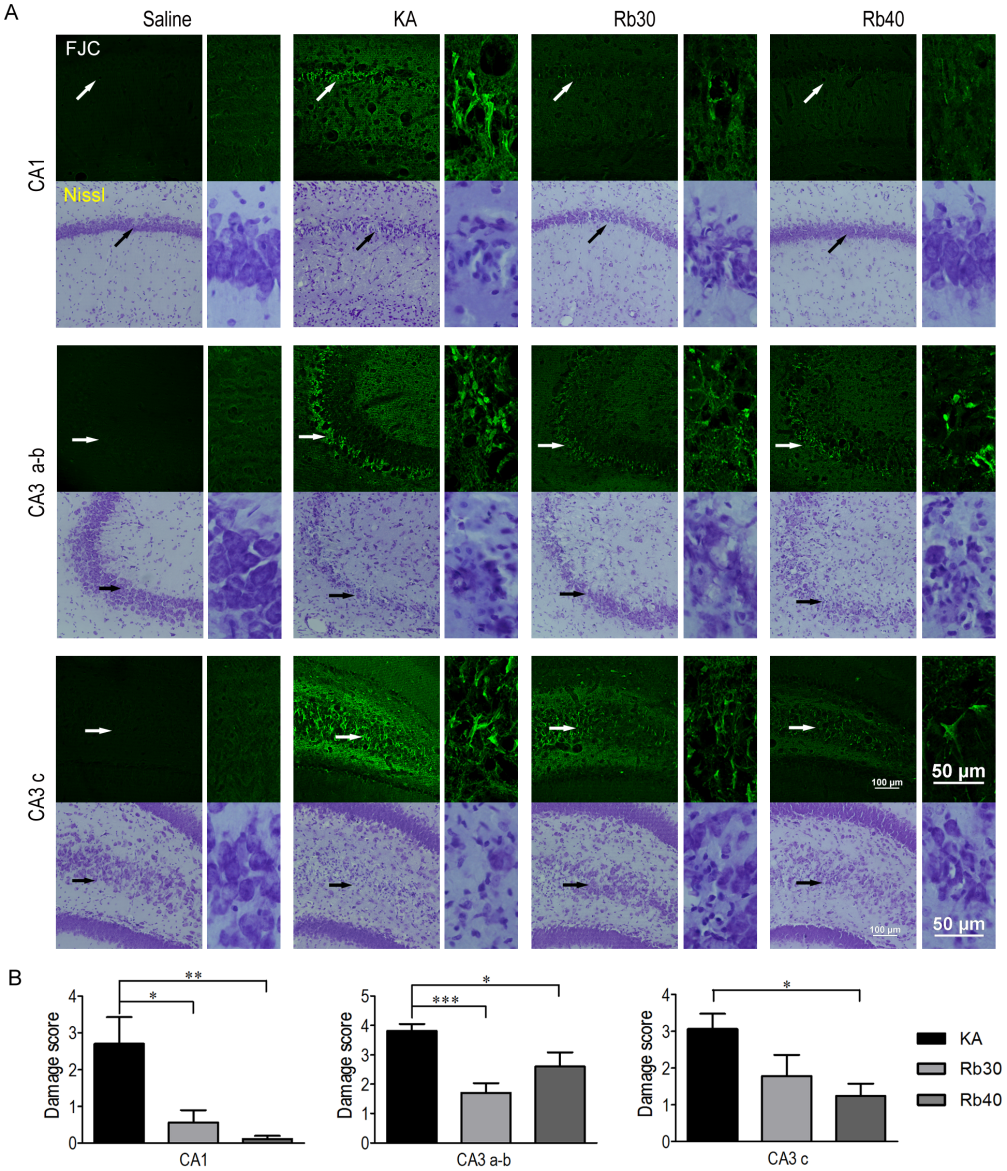
Rb fraction attenuates KA-induced acute degeneration in hippocampal pyramidal neurons. Rats were pretreated with saline as KA controls, Rb fraction 30/kg (Rb30) or 40 mg/kg (Rb40) followed 40 min later by icv KA injection, and saline controls (Saline) received icv saline injection. After 48 h, brains were removed and stained for FJC and cresyl violet. (A) Representative images (200×) of the hippocampal subfields of CA1, CA3a–b, and CA3c stained with FJC or cresyl violet (Nissl staining). Images with higher magnification (400×) showed the details of pyramidal neurons (arrowhead) in each subfield. (B) Damage scores of the three subfields of hippocampus were measured in the sections stained with FJC in each experimental group. Data are expressed as means ± SEM; n  =  6–8 per group; *p < 0.05, **p < 0.01, ***p < 0.001 by a one-way analysis of variance followed by Dunnet's post-test.

In order to choose the animal to undergo status epilepticus for the determination of the effect of Rb fraction on KA-induced neuronal damage, seizure activity following KA injection was compared across the animals pretreated with saline or Rb fraction at 30 mg/kg or 40 mg/kg after KA administration. Eight out of ten in the KA control group (KA), six out of ten in the Rb 30 mg/kg group (Rb30), and seven out of ten in the Rb 40 mg/kg group (Rb40) had a status epilepticus (stage 3 or higher seizures persistent for 4 hours). No animal was dead in the groups following KA. Given that seizure severity could be related to the degree of neuronal loss after KA injection, thus interrupting the assay/evaluation of a potential neuroprotective effect of Rb fraction, the animals that experienced status epilepticus were used for the later histological analysis in all the experimental groups (Fig. 2A). The animals that only had mild seizures (lower than stage 3 seizures, or brief stage 3 and higher stage seizures) or the body weight loss lower than 10% were excluded. Additionally, all the animals that had experienced status epilepticus had 10% or higher body weight loss at 24 or 48 hours after KA injection (Fig. 2A). To determine whether Rb fraction is able to produce seizure-independent neuroprotection against acute excitotoxicity after KA administration, degenerating neurons and surviving neurons in the hippocampus were compared between the groups of saline controls (Saline), KA controls (KA), and Rb fraction pretreatment at 30 mg/kg (Rb30) or 40 mg/kg (Rb40) 48 hours after KA. As showed in Fig. 3, saline controls (Saline) showed normal histology of neurons in hippocampal subfields, while the animals treated with KA alone (KA) had robust degenerative neurons as indicated by strong FJC staining (Fig. 3A) with great damage scores (Fig. 3B) in stratum pyramidal of hippocampal CA1, CA3a–b, and CA3c subfields. Consistently, Nissl staining showed massive cell loss and fewer surviving (intact) neurons in these areas (Fig. 3A). DG granule cells were not injured by KA. Pretreatment with Rb fraction at 40 mg/kg 40 min before KA significantly decreased KA-induced neurodegeneration in all hippocampal subfields, as indicated by the higher number of surviving neurons identified by Nissl staining and much weaker FJC positive staining (Fig. 3A) with lower damage scores when compared to KA controls (*p* < 0.05, *p* < 0.01). Rb fraction at 30 mg/kg had a similar neuroprotective efficacy as showed by Nissl staining and by FJC staining (Fig. 3A), which indicated a significant decrease in neuronal damage scores in CA1 and CA3 subfields (*p* < 0.05, *p* < 0.001 versus KA) (Fig. 3B).

### Rb fraction prevents KA-induced acute loss of GABAergic interneurons in the hippocampus

**Figure 4:**
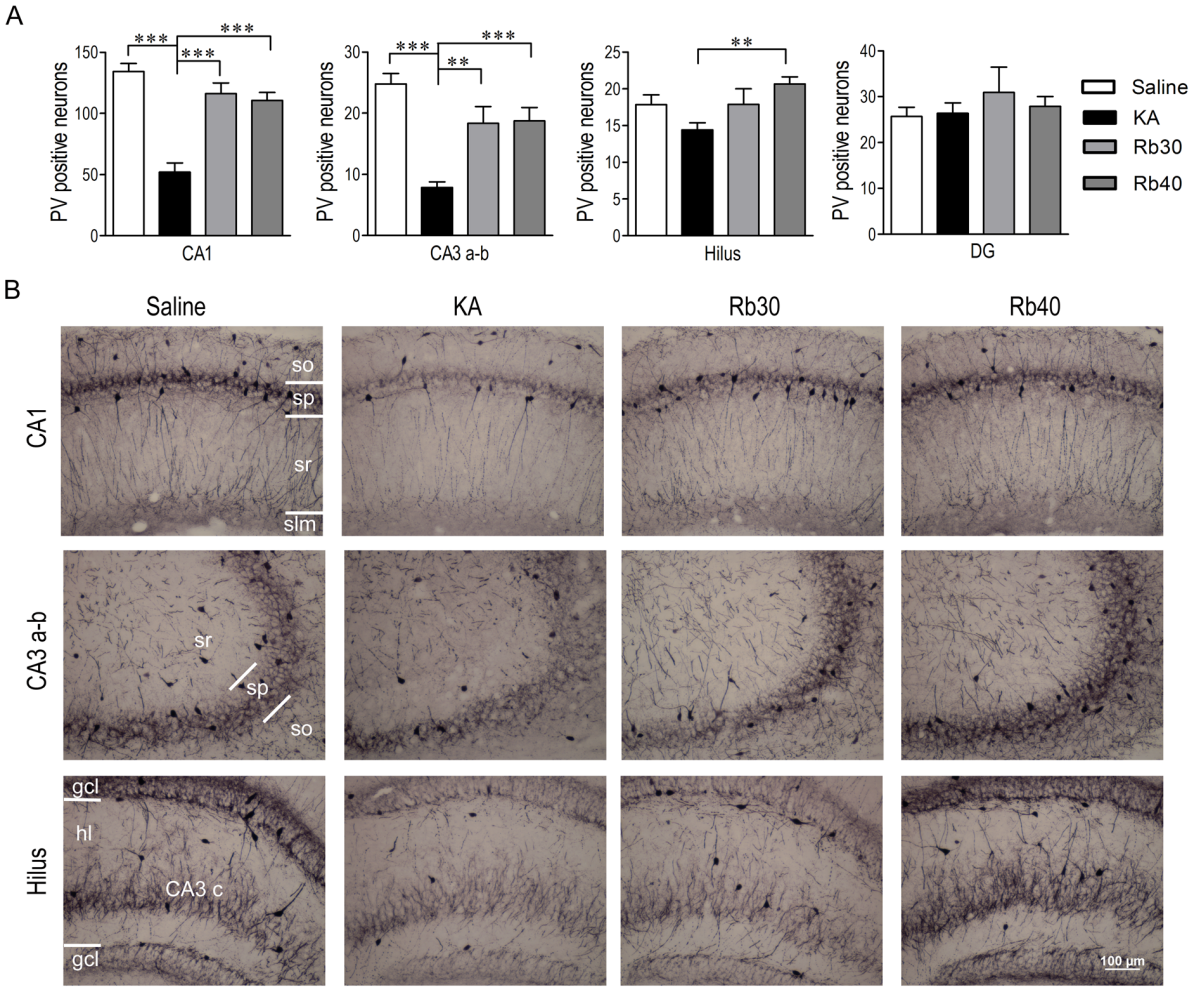
Rb fraction prevents KA-induced acute loss of PV+ interneurons in the hippocampus. Rats were pretreated with saline as KA controls, Rb fraction 30/kg (Rb30) or 40 mg/kg (Rb40) for 40 min followed by icv KA injection, and saline controls (Saline) received icv saline injection. After 48 h, brains were removed and PV+ interneurons were identified by immunohistochemical staining. (A) Numbers of PV+ interneurons in the hippocampal subfields of CA1, CA3a-b, hilus (including CA3c), and DG. (B) Representative photomicrographs (200×) of the CA1, CA3 a-b, and hilus subfields stained with anti-PV in the experimental groups. Data are expressed as means ± SEM; n  =  6–8 per group; **p < 0.01, ***p < 0.001 by a one-way analysis of variance followed by Dunnet's post-test.

**Figure 5:**
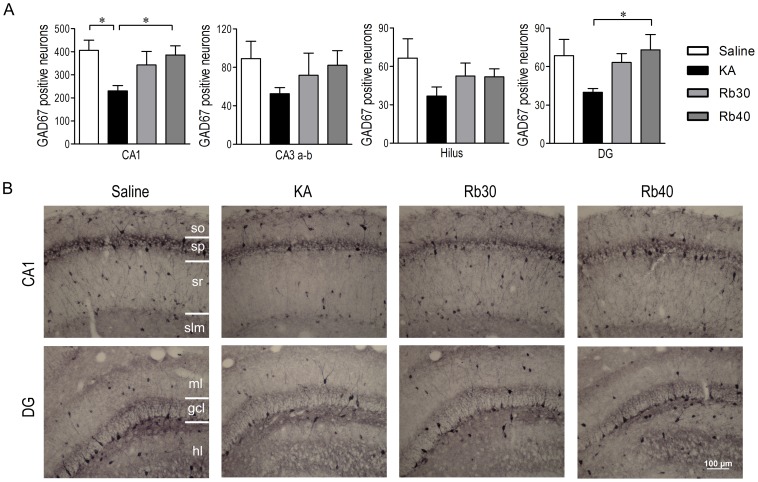
Rb fraction prevents KA-induced acute loss of GAD67+ interneurons in the hippocampus. Rats were pretreated with saline as KA controls, Rb fraction 30/kg (Rb30) or 40 mg/kg (Rb40) for 40 min followed by icv KA injection, and saline controls (Saline) received icv saline injection. After 48 h, brains were removed and GAD67+ interneurons were identified by immunohistochemical staining. (A) Quantification of GAD67+ interneurons in the hippocampal subfields of CA1, CA3a-b, hilus (including CA3c), and DG. (B) Representative photomicrographs (200×) of the CA1 and DG subfields stained with anti-GAD67 in each experimental group. Data are expressed as means ± SEM; n  =  6-8 per group; *p < 0.05 by a one-way analysis of variance followed by Dunnet's post-test.

Given the important role GABAergic interneurons play in hippocampal function and their vulnerability to excitotoxicity, we needed to determine whether Rb fraction can protect GABAergic interneurons against KA-induced excitotoxicity. The numbers of GAD67+ or PV+ interneurons were compared between the experimental groups 48 hours after KA. As shown in Fig. 4 and Fig. 5, saline controls had considerable GAD67+ or PV+ cell bodies with abundant processes in hippocampal subfields of CA1, CA3a-b and hilus. In contrast, 48 hours after KA administration, the number of PV+ positive cells significantly decreased in the hippocampal subfields CA1 and CA3a–b and showed a tendency to decrease in the hilus (including CA3c). However, the number of cells in DG was not affected in the KA group compared to Saline group (Fig. 4A). In these KA alone-treated animals, the number of GAD67+ neurons decreased only in CA1 subfield and showed a tendency to decrease in the other areas of the hippocampus (Fig. 5A) as compared to saline controls. The data suggest that PV+ neurons or PV protein level may be more vulnerable to KA than GAD67+ neurons or GAD67 protein level. The pretreatment with Rb fraction at 30 mg/kg or 40 mg/kg maintained GAD67+ cell numbers to a level similar to the saline group in all the measured areas (Fig. 5A). Similarly, the Rb pretreatment at 30 mg/kg or 40 mg/kg significantly prevented KA-induced decreases of PV+ cells in CA1, CA3a-b, and hilus (Fig. 4A). The data indicated that Rb fraction can protect GABAergic interneurons against KA-induced acute excitotoxicity.

### Rb fraction prevents KA-induced hippocampal-dependent spatial memory impairment

Here, we want to know whether the neuroprotection produced by Rb fraction during the acute period after KA can produce a beneficial outcome for cognitive function. For this purpose, we used the Morris water maze task to compare cognitive abilities, which are known to depend on the hippocampal function, between the experimental groups 16 days after KA administration. This task contained the training test and probe trial. During the training test, animals learned to locate a submerged platform in a pool by orienting themselves using distal visual cues, and the latency to find the platform negatively correlated to learning ability. During the probe trial, the platform was removed, and the number of times the animal crossed the target quadrant reflected memory ability. To avoid the potential interruption from acute seizures to the interpretation of experimental results, animals chosen for all the experiments experienced similar seizure severity and body weight loss at 24 or 48 h after KA in all the groups (Fig. 2B).

**Figure 6:**
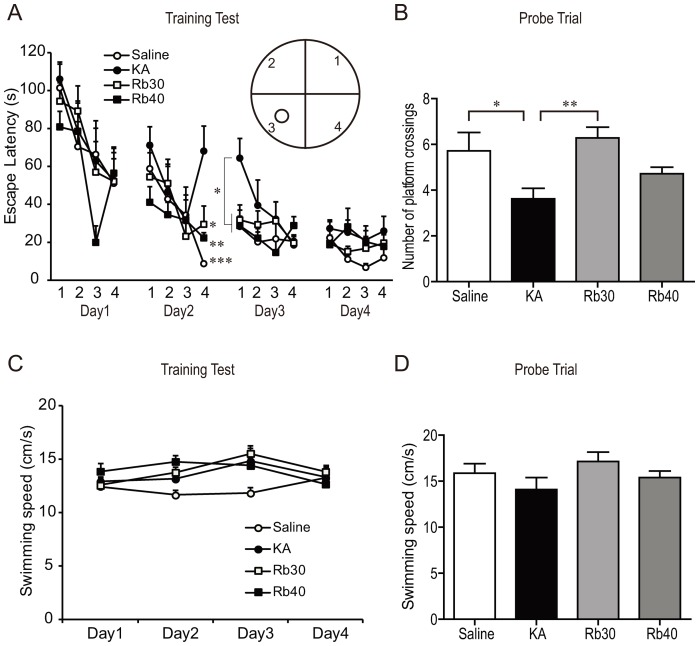
Rb fraction prevents KA-induced hippocampal-dependent spatial memory impairment. Rats received a single injection of saline as KA controls, Rb (30 mg/kg) or Rb (40 mg/kg) 40 min before icv KA administration, and saline controls (Saline) received icv saline injection. Sixteen days later, the Morris water maze task was conducted. (A) Escape latency (the time to reach the platform) in the training test. (B) The numbers of times rats crossed the area where the platform was located in the probe trial. (C) Swimming speed of rats during the training test. (D) Swimming speed of rats in the probe trial. Data are expressed as means ± SEM; n  =  6–8 per group; *p < 0.05, **p < 0.01, ***p < 0.001 by a one-way analysis of variance followed by Dunnet's post-test. Data of escape latency were analyzed by a two-way analysis of variance with repeated measures.

KA alone injected rats showed impairment of memory, and this impairment was significantly prevented by the Rb pretreatment. During the training test of four days, animals treated with KA alone had a longer escape latency to reach the hidden platform in the fourth trial of the 2^nd^ day and the first trial of the 3^rd^ day, and a tendency to a longer escape latency in the first trial of the 2^nd^ day when compared to Saline group and Rb fraction pretreated groups (*p* < 0.05, *p* < 0.01, *p* <0.001, Fig. 6A). The KA treated animals did not differ behaviorally with those of the saline controls in the other trials of the 3^rd^ day and in the four trials of the 4^th^ day. These data indicated that while animals treated with KA alone were able to learn the task, they did not perform as well as the controls, implying that these animals had difficulties in either retrieving the stored memory and/or forming a strong long-term memory regarding the position of the platform at the beginning of the training procedure. In contrast, animals pretreated with Rb fraction at 30 mg/kg or 40 mg/kg had similar escape latency to that of saline controls in all the trials (Fig. 6A), suggesting that the animals treated with Rb fraction before KA maintained a normal ability to learn and retrieve the stored memory. Consistent results were obtained from the probe trial. Specifically, KA alone-treated animals showed memory deficit because these animals significantly decreased the number of times they crossed the area where the platform was located during the training test compared to Saline group or the Rb 30 mg/kg pretreatment group (*p* < 0.05, *p* < 0.01, Fig. 6B). Rb fraction produced protective effect against KA-induced memory deficit as indicated by the fact that animals pretreated with Rb fraction (30 mg/kg) crossed the area where the platform was located during the training test more frequently compared with the KA controls, and rats pretreated with 40 mg/kg of Rb fraction showed a strong tendency to cross the area where the platform was located during the training test more frequently compared with the KA controls. The results from the swimming speed test showed no significant differences between the different groups both in the training test and the probe trial (*p* > 0.05, Fig. 6C, D), indicating that there was no difference in physical strength between the different groups. Taken all together, the data demonstrated that Rb fraction as a pretreatment can prevent KA-induced impairment of hippocampal-dependent spatial cognitive function during the chronic period after KA administration.

### Rb fraction prevents chronic hippocampal neurodegeneration after KA injection

To explore the possibility that Rb fraction maintains its early neuroprotection in the hippocampus against chronic KA-induced neurodegeneration and consequently prevents the impairment of hippocampal-dependent cognitive function, the numbers of dying pyramidal neurons and surviving GABAergic neurons in the hippocampus were compared between KA controls and Rb fraction-pretreated animals after the Morris water maze task.

**Figure 7:**
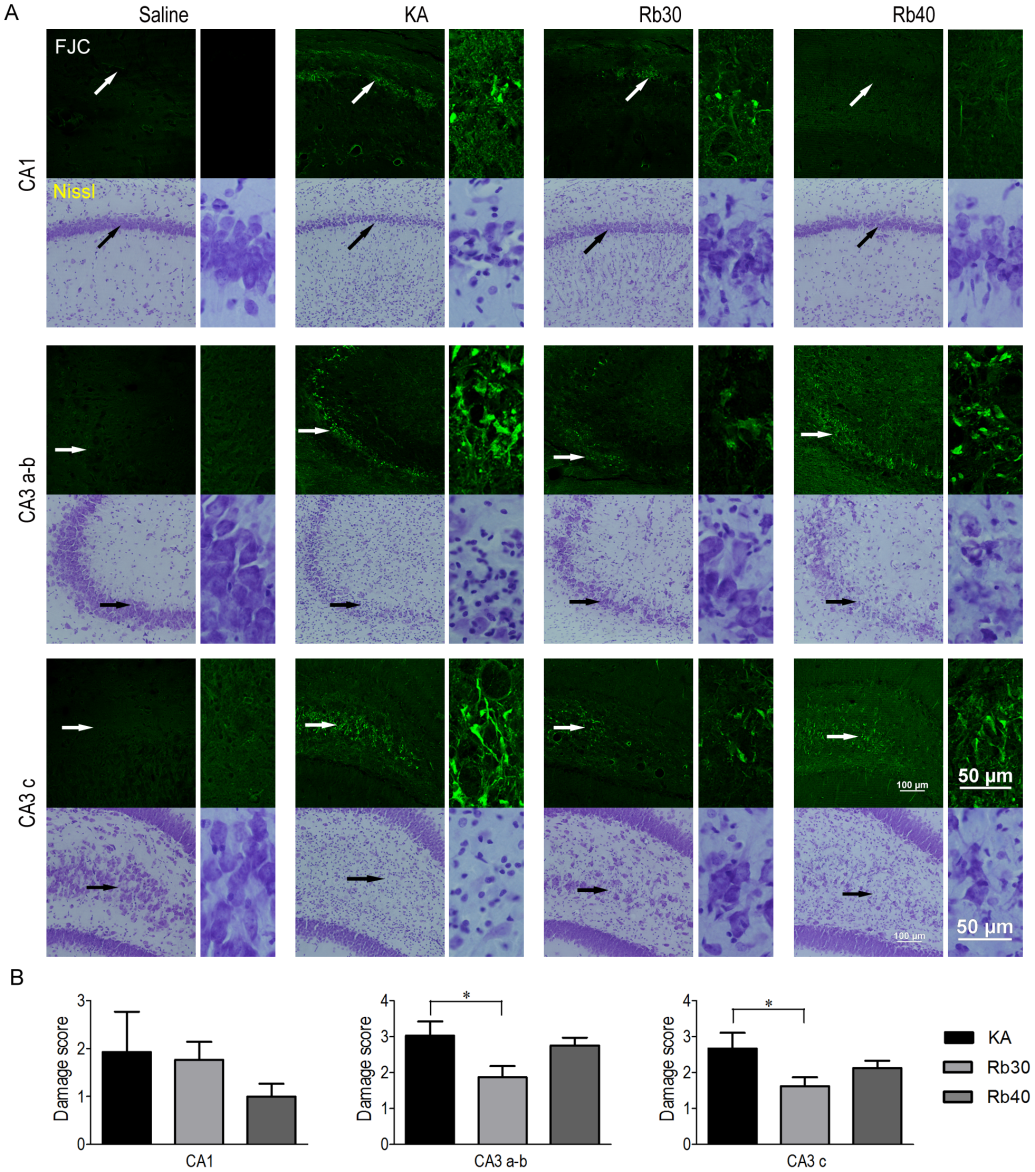
Rb fraction decreases KA-induced hippocampal pyramidal neurodegeneration during chronic period after KA injection. Rats received a single injection of saline as KA controls, Rb (30 mg/kg) or Rb (40 mg/kg) 40 min before icv KA administration, and saline controls (Saline) received icv saline injection. After the Morris water maze task (3 weeks after KA), brains were removed and stained for FJC or cresyl violet (Nissl staining). (A) Representative images (200×) of the hippocampal subfields of CA1, CA3a–b, and CA3c stained with FJC or cresyl violet. Images with higher magnification (400×) showed the details of pyramidal neurons (arrowhead) in each subfield. (B) Damage scores of the three subfields of hippocampus were measured in each experimental group. Data are expressed as means ± SEM; n  =  6–8 per group; *p < 0.05, **p < 0.01 by a one-way analysis of variance followed by Dunnet's post-test.

Consistent with the acute period (48 hours) after KA, the hippocampal subfields of CA3 and CA1 in KA-treated animals 3 weeks after KA injection showed abundant dying pyramidal neurons identified by FJC staining (Fig. 7). That suggests a long-lasting pyramidal neurodegeneration in KA-treated rats. When compared to the KA controls, animals pretreated with Rb fraction at 30 mg/kg showed a significant decrease in the number of dying pyramidal neurons in the areas of CA3a-b and CA3c but not in CA1, which accompanied considerable surviving pyramidal neurons in CA3 subfield identified by Nissl staining. The pretreatment with Rb fraction at 40 mg/kg only had a strong tendency towards protecting the pyramidal neurons in CA1 as indicated by FJC staining and Nissl staining. These data suggested that Rb fraction at 30 mg/kg was more effective than at 40 mg/kg for the prevention of delayed neuronal death in the hippocampal pyramidal neurons following KA excitotoxicity.

**Figure 8:**
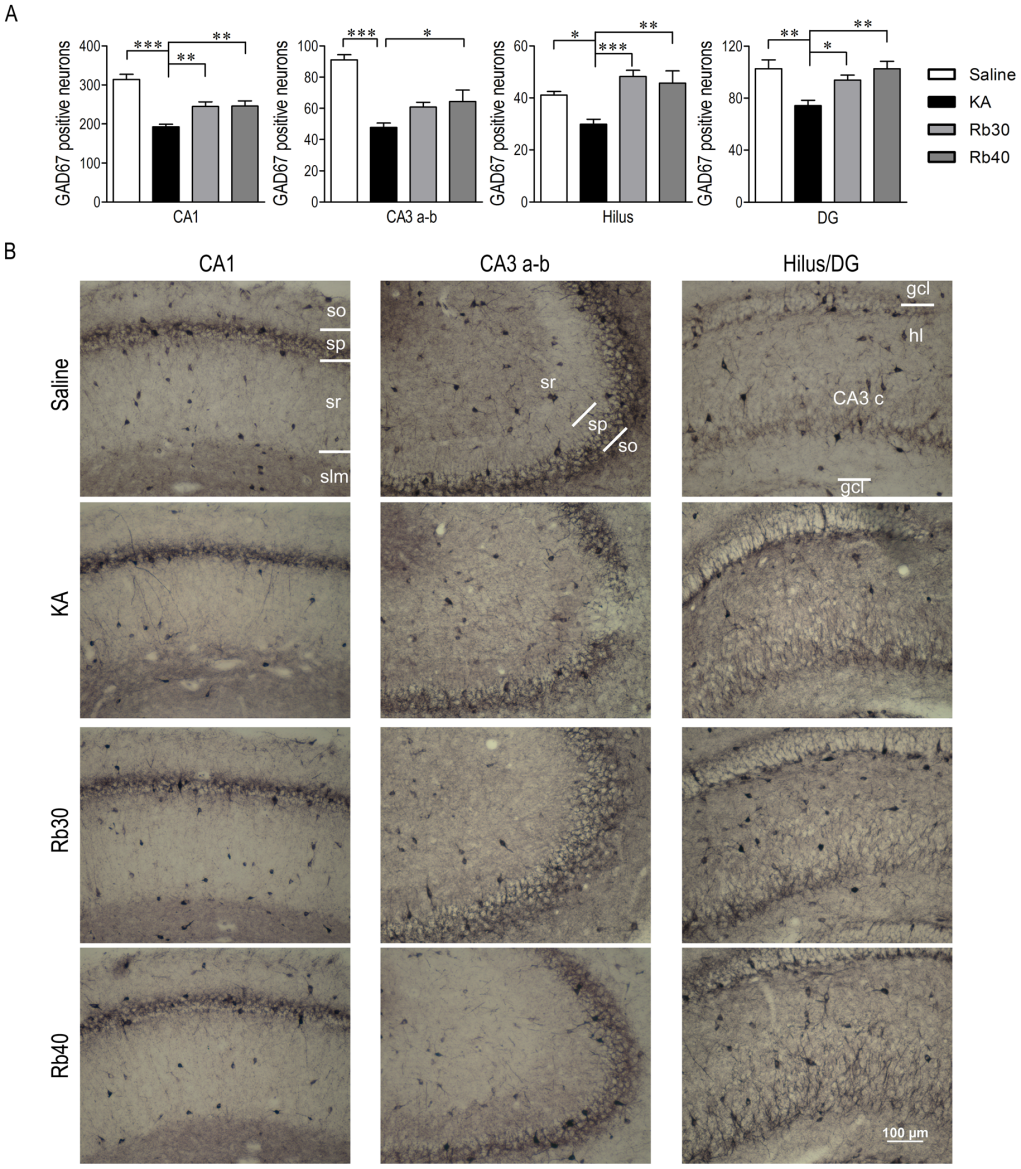
Rb fraction decreases KA-induced loss of hippocampal GAD67+ interneurons during chronic period after KA injection. Rats received a single injection of saline as KA controls, Rb (30 mg/kg) or Rb (40 mg/kg) 40 min before icv KA administration, and saline controls (Saline) received icv saline injection. After the Morris water maze task (3 weeks after KA), hippocampal GAD67+ interneurons were detected by immunohistochemical staining. (A) Numbers of GAD67+ interneurons in the hippocampal CA1, CA3a–b, hilus (including CA3c), and DG regions. (B) Representative photomicrographs (200×) of the CA1, CA3a–b, and hilus in the experimental groups. Data are expressed as means ± SEM; n  =  6–8 per group; *p < 0.05, **p < 0.01, ***p < 0.001 by a one-way analysis of variance followed by Dunnet's post-test.

**Figure 9:**
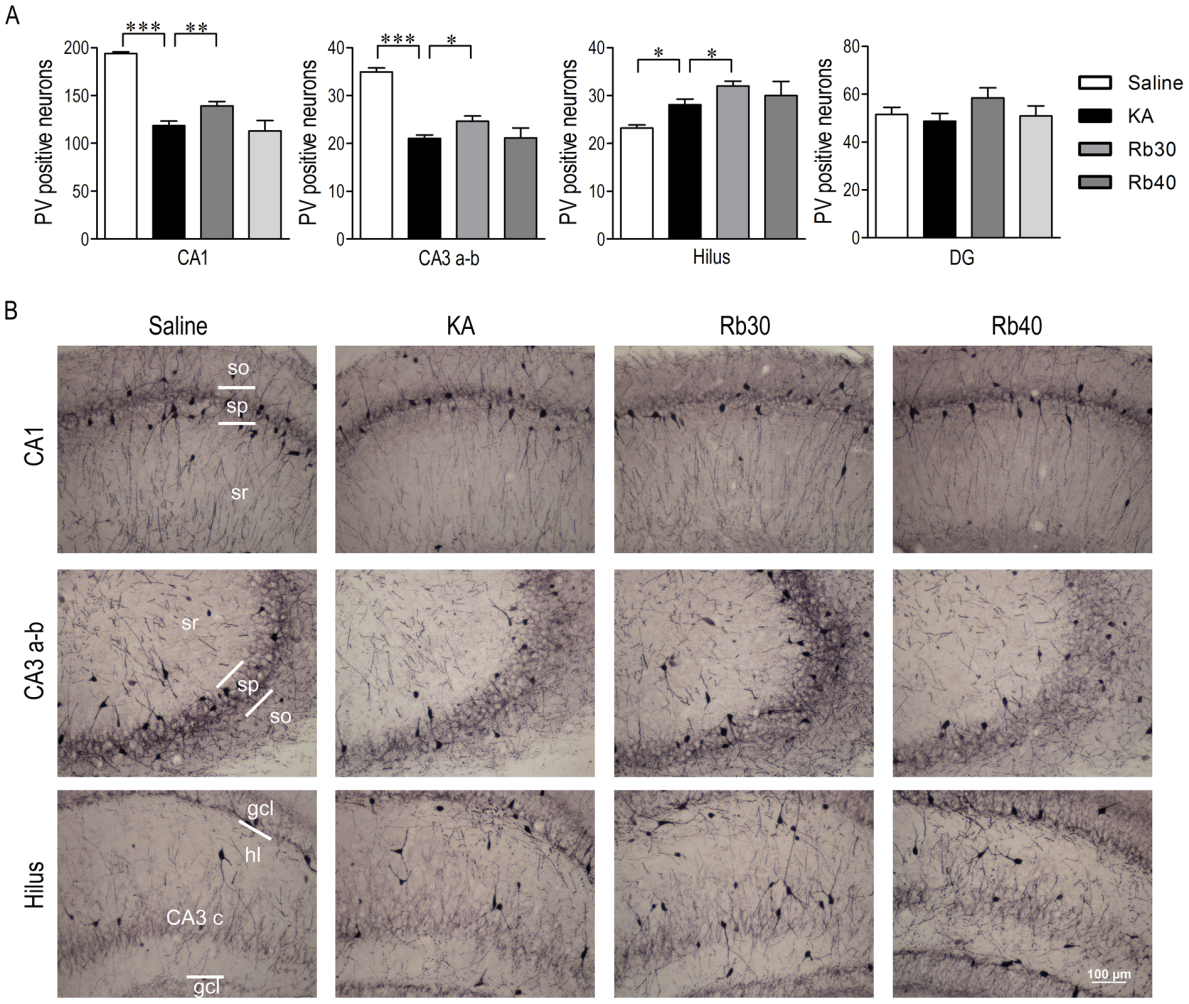
Rb fraction decreases KA-induced loss of hippocampal PV+ interneurons during chronic period after KA injection. Rats were pretreated with saline as KA controls, Rb fraction 30/kg (Rb30) or 40 mg/kg (Rb40) followed 40 min later by icv KA injection, and saline controls (Saline) received icv saline injection. After the Morris water maze task (3 weeks after KA), brains were removed and hippocampal PV+ interneurons were identified by immunohistochemical staining. (A) Numbers of PV+ interneurons in the hippocampal subfields of CA1, CA3a–b, hilus (including CA3c), and DG. (B) Representative photomicrographs (200×) of the CA1, CA3 a–b, and hilus in the experimental groups. Data are expressed as means ± SEM; n  =  6–8 per group; *p < 0.05, **p < 0.01, ***p < 0.001 by a one-way analysis of variance followed by Dunnet's post-test.

Immunohistochemistry assay showed a long-lasting protective effect of Rb fraction on hippocampal GABAergic interneurons (Fig. 8 and Fig. 9). Three weeks after KA, GAD67+ cells significantly decreased in all measured areas including CA1, CA3a-b, hilus, and DG (*p* < 0.05, *p* < 0.01, *p* < 0.001 vs. Saline, Fig. 8A), while 48 hours after KA, GAD67+ cells decreased only in CA1 area (*p* < 0.05 vs. Saline). This demonstrated that GAD67+ cells further degenerated during the chronic period after KA, indicating a delayed injury of GAD67+ cells. Consistent with the acute period after KA (Fig. 4), PV+ interneurons significantly decreased in CA1 and CA3a–b (*p* < 0.001 vs. Saline, Fig. 9A) and were not affected in DG compared to saline controls. However, the number of PV+ interneurons in hilus tended to change from a decrease to an increase in the chronic period, perhaps indicating a compensatory increase due to neural regeneration in this area. Interestingly, the protective effect of Rb fraction on GABAergic interneurons during the chronic period was more potent than during the acute period. Specifically, Rb fraction at both doses significantly increased GAD67+ cell numbers in most measured areas (except for CA3a–b at a dose of 30 mg/kg) during the chronic period, but only the higher dose was effective during the acute period (Fig. 5). Furthermore, Rb fraction at 30 mg/kg significantly increased the number of PV+ cells in CA1, CA3a–b, and hilus (including CA3c) when compared to KA controls, but the higher dose was not effective (Fig. 9). These data indicated that Rb fraction at 30 mg/kg was more effective than at 40 mg/kg, which is consistent with the better efficacy of 30 mg/kg of Rb fraction in the protection of hippocampal pyramidal neurons and cognitive function during the chronic period after KA excitotoxicity (Fig. 6, Fig. 7). Additionally, the Rb pretreatment maintained the numbers of GAD67+ cells in DG and hilus and PV+ cells in hilus to the same level of saline controls (Fig. 8A and Fig. 9A). Altogether, the data demonstrated that Rb fraction can produce a long-lasting protection of hippocampal pyramidal neurons, especially GABAergic interneurons, against KA excitoxicity, and consequently prevent the KA-induced impairment of hippocampal-dependent cognitive function.

### Rb fraction protects astrocytes and inhibits microglial activation against KA excitotoxicity

**Figure 10:**
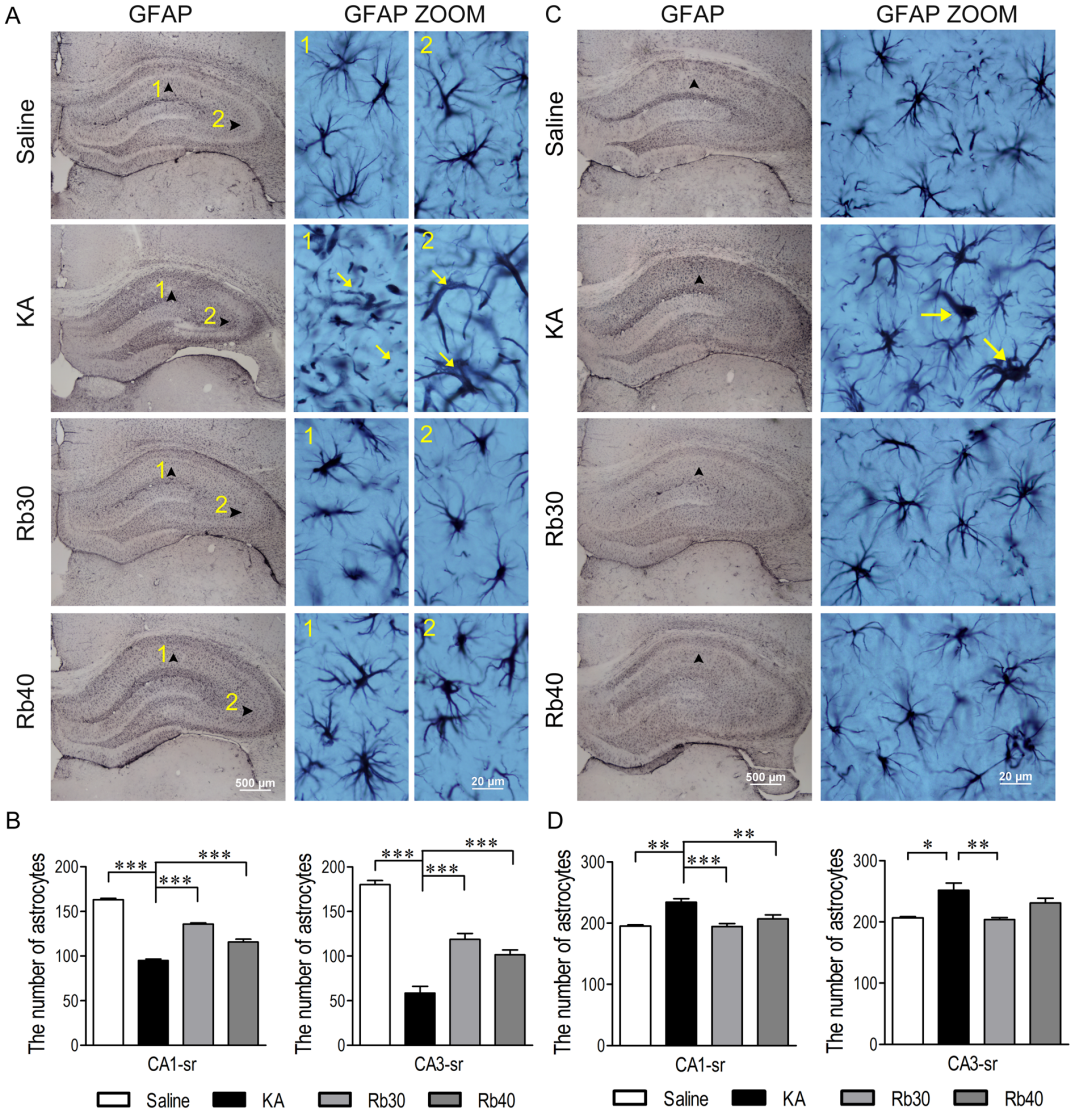
Rb fraction protects astrocytes during acute period and attenuates astrogliosis during chronic period after KA injection. Rats were pretreated with saline as KA controls, Rb fraction 30/kg (Rb30) or 40 mg/kg (Rb40) followed 40 min later by icv KA injection, and saline controls (Saline) received icv saline injection. After 48 h or 3 weeks, brains were removed, and astrocytes in the hippocampus were detected by GFAP immunostaining. Images (40×) showed the distribution of astrocytes 48 h (A) or 3 weeks (C) after KA administration, the morphology of cells in the CA1 or CA3 stratum radiatum (arrowhead) were showed at high magnification (1000×). Numbers of astrocytes in the hippocampal subfields of CA1 stratum radiatum and CA3 stratum radiatum 48 h (B) or 3 weeks (D) after KA injection were determined and averaged across animals in each experimental group. Data are expressed as means ± SEM; n  =  6–8 per group; **p < 0.01, ***p < 0.001 by a one-way analysis of variance followed by Dunnet's post-test.

Astrocytes and microglia have been recognized to be involved in KA-induced acute and delayed neuronal damage. Therefore, we next determined whether Rb fraction could affect the responses of astrocytes and microglia during both acute and chronic periods after KA injection. During the acute period (48 hours after KA), immunohistochemistry assay indicated that astrocytes, depending on the areas in the hippocampus, responded differently to KA-induced acute excitotoxicity (Fig. 10A). Astrocytes in CA1 stratum radiatum were severely damaged as evident by the fact that astrocytes had a fragmented appearance characteristic of dying cells (arrowhead in Fig. 10A KA-1). In some animals, swollen astrocytes with increased GFAP expression and profoundly thickened and fewer processes could be detected in CA3 stratum radiatum (arrowhead in Fig. 10A KA-2), but the number of GFAP+ cells significantly decreased in both these areas compared to that in saline controls (Fig. 10B), indicating a loss of astrocytes or an overall decrease in GFAP level in these two areas 48 hours after KA. Interestingly, during the chronic period after KA (3 weeks later), the injured astrocytes that initially appeared during the acute period disappeared. Instead, astrogliosis occurred as astrocytes were observed to increase in number, size, length, and thickness of their processes compared to the saline controls in the entire hippocampus of both hemispheres as shown in Fig. 10C, D. This demonstrated that astrocytes were vulnerable to KA-induced acute excitotoxicity in the hippocampal subfields CA1 and CA3, suggesting a loss of the neuroprotection provided by astrocytes against KA-induced excitotoxicity. The pretreatment with Rb fraction at 30 or 40 mg/kg profoundly protected astrocytes against KA-induced acute excitotoxicity. This was shown by the fact that Rb fraction significantly attenuated KA-induced decrease in GFAP+ cells and by the fact that the intact cell body and their process in Rb groups were similar to those in saline controls (Fig. 10C, D). Notably, the pretreatment with Rb fraction at both doses, particularly at 40 mg/kg, maintained some activation of astrocytes at 48 hours after KA injection compared to saline controls as indicated by the more intense GFAP staining in cell body and process in the rats pretreated with 40 mg/kg than in saline controls (Fig. 10A, Rb40). While the astrocytic activation by Rb fraction that initially appeared during the acute period disappeared 3 weeks later, the number of GFAP+ cells and the morphology of astrocytes were not significantly different from ones in saline controls (Fig. 10C, D). The data demonstrated that Rb fraction can protect astrocytes against KA-induced excitotoxicity and thus could maintain the neuroprotective functions of astrocytes against KA-induced acute excitotoxicity.

**Figure 11:**
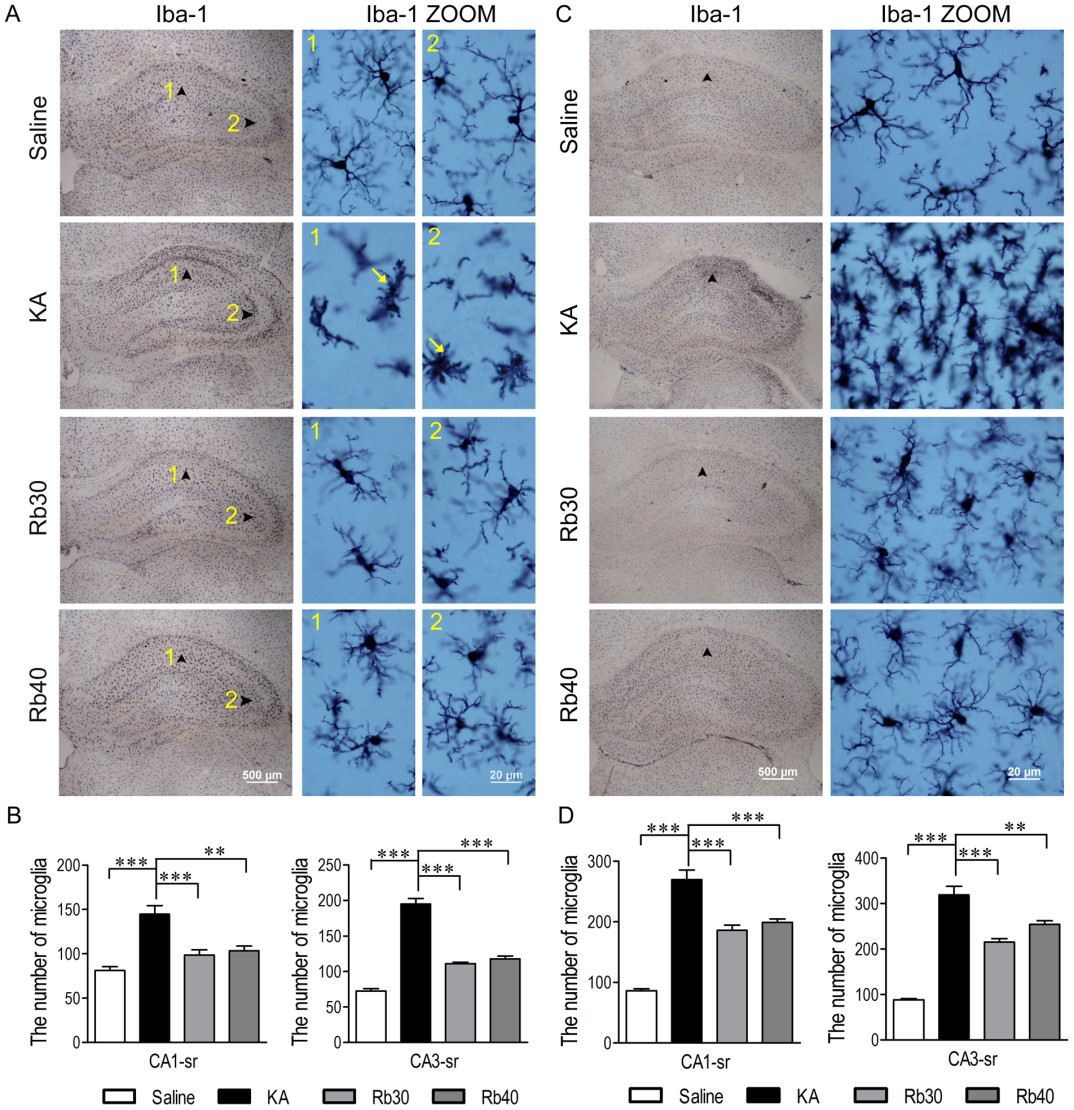
Rb fraction inhibits the activation of microglia after KA injection. Rats were pretreated with saline as KA controls, Rb fraction 30/kg (Rb30) or 40 mg/kg (Rb40) followed 40 min later by icv KA injection, and saline controls (Saline) received icv saline injection. Forty-eight hours or 3 weeks later, brains were removed, and microglia were detected by Iba-1 immunostaining. Images (40×) showed the distribution of microglia in the hippocampus 48 h (A) or 3 weeks (C) after KA administration, the morphology of cells in CA1 or CA3 stratum radiatum (arrowhead) were showed at high magnification (1000×). Numbers of microglia in the hippocampal subfields of CA1 stratum radiatum and CA3 stratum radiatum 48 h (B) or 3 weeks (D) after KA injection were determined and averaged across animals in each experimental group. Data are expressed as means ± SEM; n  =  6–8 per group; *p < 0.05,**p < 0.01, ***p < 0.001 by a one-way analysis of variance followed by Dunnet's post-test.

In comparison to the diverse responses of astrocytes to KA-induced excitotoxicity, microglia were universally activated in the hippocampus after KA injection and activated microglial cells continued to proliferate throughout the duration of the study (Fig. 11). Activated microglial cells with big cell bodies, short and thick processes, which were intensely stained for Iba-1, appeared in all the layers of the hippocampus of both hemispheres in the rats treated with KA alone at 48 hours after KA injection (Fig. 11A). Three weeks later, the number of activated microglial cells further increased significantly, the cell bodies were enlarged, and their cellular processes became shorter and thicker (Fig. 11B-D). Rb fraction pretreatment at 30 or 40 mg/kg significantly decreased KA-induced activation of microglia throughout the duration of the study as indicated by the similar density of cell body and the similar morphology of microglia in cell body and their processes to normal ones at 48 h and 3 weeks after KA injection (Fig. 11B, D). Particularly, at the later time, cell body density markedly decreased in Rb-pretreated rats compared to that in KA controls and maintained a similar level as that of the saline controls (Fig. 11C, D). Given the activation of microglia responsible for delayed neurodegeneration after KA injection, the above data indicate that the inhibition of microglial activation by Rb fraction may have contributed to the neuroprotection against delayed neuronal injury in GABAergic interneurons provided by Rb fraction following KA administration.

## Discussion

Decades of pathological and physiological studies have been focused on neurons in neurodegenerative disorders, but it is becoming increasingly evident that glial cells also play a critical role in brain homeostasis and synaptic plasticity [36]. Specifically, astrocyte loss or dysfunction, microglial activation, and neuron death or degeneration (particularly that of GABAergic interneuron) always occur in acute and chronic neurological disorders. More importantly, these events intersect with each other, which creates a vicious cycle, and can contribute to brain injuries like trauma or stroke or neurodegenerative disorders (including Alzheimer's disease, Parkinson disease, epilepsy and seizures). Our previous studies demonstrated that the purified PDS extract, made up of the three individuals of PDS, is protective for motor function in 3-nitropropionic acid (an inhibitor of succinate dehydrogenase)-induced neurodegeneration that models Huntington's disease [28], and for the principal neurons of the hippocampus in a seizures-independent manner following KA injection [30]. The present study further demonstrated that a highly purified PDS fraction (Rb fraction), consisting of the five individuals of PDS, is protective against KA-induced impairment of hippocampus-dependent cognitive function, and the protected principal neurons and GABAergic interneurons in the hippocampus by Rb fraction together contribute to this functional protection. Furthermore, this study also suggests that Rb fraction can maintain glial homeostasis despite the disruption of KA-induced excitotoxicity because Rb fraction significantly protected hippocampal astrocytes and prevented microglial activation in the hippocampus following KA administration. This homeostasis may contribute to the neuroprotective activity of Rb fraction against KA-induced acute and delayed neurodegeneration. The data obtained from this study indicates that Rb fraction is promising for the prevention and treatment of neurodegenerative diseases.

Rb fraction protected principal neurons and GABAergic interneurons in the hippocampus and thus can protect hippocampus-dependent cognitive function against KA-induced excitotoxicity. KA can elicit selective neuronal death in many regions of brain, particularly in the hippocampal subregions of CA1 and CA3, and in the hilus of DG, in which the pathological changes partially mimic neurodegeneration in the CNS [11], [37]. GABAergic interneurons, particularly PV+ interneurons and GAD67+ interneurons, are highly susceptible to KA toxicity [4], [6], [13]. Consistently, cognitive deficits following KA-induced excitotoxicity were reported previously [38], [39]. This study demonstrated that Rb fraction can provide a long-lasting protection of both hippocampal pyramidal neurons and GAD67+ or PV+ interneurons and also hippocampus-dependent cognitive protection against KA toxicity. There are several sets of evidence to support the contributive role of the long-lasting protection of both hippocampal pyramidal neurons and GAD67+ or PV+ interneurons produced by Rb fraction for the cognitive protection against KA-induced excitotoxicity. First, hippocampal network oscillations generated locally as a result of reciprocal interactions between excitatory neurons (principle neurons) and inhibitory interneurons (GABAergic interneurons) [40]-[43] is crucial for cognitive function [44], [45]. Second, hilar GABAergic interneuron plays a critical role in controlling spatial learning and memory retrieval [22]; impairment of hilar GABAergic interneurons leads to learning and memory deficits in AD mice, and treatment with the GABA(A) receptor potentiator pentobarbital alleviates the learning and memory deficits [18]. Consistently, in the present study, the animals treated with KA alone showed an impairment of spatial memory retrieval, and Rb fraction protected the hilar GABAergic interneurons and the impairment of spatial memory retrieval following KA-induced excitotoxicity. Moreover, the balance of excitatory and inhibitory neuronal activity in the hippocampus is thought to be required for normal learning and memory [46], while an imbalance due to the loss of hippocampal GABAergic interneurons including GAD67+ cells and PV+ cells has been implicated in the pathogenesis of cognitive impairment in Alzheimer's disease (AD) [18], [47]. Particularly, among the subpopulations of GABAergic interneurons, PV+ cells generated the oscillations [48], [49] that are required for hippocampal function and associated behavior [50], [51], so their dysfunction or loss links reduced gamma oscillatory activity and cognitive deficit in AD mice [22], [52], [53].

Rb fraction could maintain glial homeostasis from the disruption of KA-induced excitotoxicity, which may partially contribute to the neuroprotective activity of Rb fraction. Reactive astrocytes and microgliosis were identified in the hippocampus after KA-induced excitotoxicity in the previous studies [14], [54]. In the present study, astrocytes were injured in the hippocampal subfields CA1 and CA3, where pyramidal neurons and GABAergic interneurons were also severely damaged at 48 hours after KA administration, during the chronic period reactive astrocytes appeared in the hippocampus. The differences in the astrocytic response to KA-induced excitotoxicity could be due to the different excitotoxic severities between the previous studies and this study. Consistent with previous studies [14], [55], [56], the present study observed that microglia were universally activated in the hippocampus at 48 hours after KA injection and activated microglial cells continued to proliferate during the chronic period. Interestingly, Rb fraction significantly protected astrocytes in CA1 and CA3 subfields against KA-induced acute excitotoxicity, which paralleled the protection of the pyramidal neurons and GABAergic interneurons provided by Rb fraction, and maintained the astrocytes at some level of activation. More strikingly, Rb fraction strongly prevented microgliosis during the chronic period after KA administration. Astrocytes have been demonstrated to support and protect neurons through multiple ways such as cleaning off extracellular glutamate, buffering extracellular ions, and providing neurons with metabolic and trophic support [1], [36]. Accordingly, astrocyte loss or dysfunction represents a potentially significant cause of neuronal degeneration [57], [58]. For example, the function of astrocytes was reported to be impaired immediately after ischemia, which was followed by an injury of GABAergic neurons [7], while the activation of astrocytes has been shown to be neuroprotective against acute neuron death but not the delayed neuron death following KA administration [59]. In comparison with astrocytes, microglial activation (microgliosis) has been proven to be responsible for the delayed hippocampal neuronal death following KA-induced excitotoxicity through production of neurotoxic mediators such as reactive oxygen species and iNOS [8], [14], [55], [56], [60]. Therefore, it is reasonable to believe that the protection of astrocytes offered by Rb fraction may partially contribute to its protection of hippocampal neurons, especially GABAergic interneurons against KA-induced acute neuron death. The attenuated microgliosis caused by Rb fraction may contribute to the long-lasting neuroprotection against the delayed neuron death following KA excitotoxicity. However, future studies are necessary to reveal the detailed mechanisms of Rb fraction to protect astrocytes and neurons, such how Rb fraction protects astrocytes and whether Rb fraction simultaneously, independently, or consequently protects astrocytes and neurons.
